# Wetlands as a solution to water browning: A 3‐year efficiency assessment of outdoor mesocosms

**DOI:** 10.1002/wer.70008

**Published:** 2025-02-04

**Authors:** Henric Djerf

**Affiliations:** ^1^ Department of Environmental Science Kristianstad University Kristianstad Sweden

## Abstract

Water browning is the phenomenon of increasing discoloration in lakes and water courses due to the leaching of organic matter from soils. This process has ecological, economic, and social implications, such as affecting light penetration, oxygen levels, nutrient cycling, drinking water quality, and recreational use. This study investigated the potential of wetlands as a natural solution to mitigate water browning by reducing organic matter and iron. Six mesocosms in pilot scale were set up and studied for 3 years for their efficiency to reduce water color, with sampling every 14th day. Parameters measured were the changes in water color, dissolved organic carbon, iron, pH, and conductivity over 3 years, under different hydrological and climatic conditions. The results showed that wetlands can decrease water browning by retaining organic matter and iron, especially in vegetated systems. This study showed that long retention times with vegetated shallow systems were needed to reduce the water color by 47%. The decrease of color was primarily due to reduction of iron that decreased by 66%, while the dissolved organic carbon (DOC) concentration decreased by only 6%. These findings highlight the potential of constructed wetlands as a valuable tool for improving water quality and mitigating the impacts of water browning, though further optimization of wetland design and integration with broader land‐use strategies is needed to address this complex issue effectively.

**Practitioner Points:**

- Constructed wetlands can mitigate brownification, especially with long retention times and shallow vegetated wetlands.
- Iron reduction is more strongly correlated with colour reduction than with DOC reduction.
- Vegetated constructed wetlands reduced the colour concentration of inflow water by 47% after a 14‐day retention time.
- Wetlands need a long retention time to reduce colour, and should be placed far upstream in the watershed.
- Vegetated systems may use alternative pathways, like biofilm formation, to reduce humic substances, independent of UV exposure.

## Graphical abstract

This mesocosm study examines the capacity of constructed wetlands (CW) to mitigate the effects of brownification by analyzing the behavior of color, iron, and dissolved organic carbon (DOC) in different CW setups during 2 year.

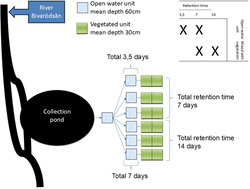

## INTRODUCTION

Lakes, streams, and rivers near peatlands are naturally colored by organic substances. In areas where mires and coniferous forests are prevalent, dystrophic lakes are commonly found. These lakes exhibit a natural brown color, a direct consequence of the substantial levels of dissolved organic matter present (Drakare et al., 2003).

The term “brownification” refers to the recent trend of increasing discoloration observed in water bodies. This browning process has been noted across numerous sites in the northern hemisphere (Kritzberg et al., 2020; Monteith et al., 2007). The coloration of water, particularly when measured at a wavelength of around 400 nm, is predominantly influenced by the presence of dissolved organic carbon (DOC) and iron (Fe) (Kritzberg & Ekström, 2012; Sarkkola et al., 2013; Xiao et al., 2013).

The discoloration of waterways, commonly referred to as “brownification,” can be attributed to a variety of factors. Prominent theories suggest that this phenomenon is due to a decrease in sulfate deposition, which then has historically limited the mobility of organic matter in soils (de Wit et al., 2016). Additionally, the widespread planting of Norway spruce, which affects soil and water chemistry, is also considered a contributing factor (Škerlep et al., 2019). The implications of climate change, such as increased precipitation and temperature fluctuations, are believed to enhance the leaching of colored organic matter into water bodies (Finstad et al., 2016). Furthermore, the drainage of peatlands for forestry or agriculture accelerates the release of DOC, intensifying water coloration (Nieminen et al., 2021). It is likely that all these factors interact and contribute to varying degrees.

Water color, despite being composed of natural substances and lacking toxicity, has a significant impact on the ecosystem (Blanchet et al., 2022). One crucial effect is related to solar radiation: Colored water (due to dissolved substances or particles) reduces the penetration of sunlight into the water column. As a result, photosynthesis occurs at shallower depths, affecting aquatic plants, for example, macrophytes (Leech et al., 2018). The consequences extend to primary production—plants and algae—which decrease due to limited sunlight caused by increased water color. Simultaneously, decomposing organisms, primarily various types of bacteria, become more prevalent, thriving on the breakdown of organic carbon (Tranvik, 1998; Tranvik & Bertilsson, 2001). Furthermore, changes in visibility depth due to water color affect the fish population. Fish species that rely on sight for hunting (e.g., *Perca fluviatilis*) are inhibited, while those using other senses (such as touch) for hunting (e.g., *Silurus glanis*) are less affected (Estlander et al., 2010). Increased water color and organic matter also cause higher expenses for drinking water treatment. According to Svenskt vatten (2021), approximately 50% of the drinking water in Sweden originates from surface water sources, which undergo various purification processes. Therefore, water color reduction is essential to ensure the quality and safety of drinking water (Klante et al., 2022). Moreover, water color influences the aesthetic and recreational value of water bodies, as clear and transparent water is perceived as “cleaner” and more attractive for activities such as swimming (Albrecht et al., 2023; Kritzberg et al., 2020).

These challenges highlight the necessity for effective mitigation strategies to manage and reduce the levels of organic matter and water color. One such approach includes the utilization of wetlands, which are known to decrease organic matter and have been employed in the treatment of sewage water (de Sá Salomão et al., 2012; Sardana et al., 2019), agricultural runoff (Vymazal & Březinová, 2018), stormwater (Svensson et al., 2015), and industrial waste water (Vymazal, 2014). Removal pathways for organic contaminants, such as physical sedimentation, plant uptake, microbial processes, and photochemical reactions, have been identified in wetlands (Sardana et al., 2019). Decomposition of organic matter is a natural process in watercourses. Research by Franke et al. (2013) highlighted biofilms in natural watercourses as critical sites for the breakdown of DOC. They noted that the extent of this breakdown is influenced by the origin and chemical makeup of the organic material, as well as the presence of essential nutrients like nitrogen and phosphorus. In addition, Andersson and Nilsson (2001) showed the significance of pH and temperature on the activities of stream microbes, by observing an increase in microbial activity in response to increased pH levels. Weyhenmeyer et al. (2014), in their study, identified flocculation, particularly between DOC and iron, as a primary process for diminishing DOC concentrations in lake environments.

Wetlands, particularly peatlands and swamps, are often contributors to downstream DOC (Aitkenhead & McDowell, 2000; Garcìa et al., 2010; Maurice et al., 2022). This has been particularly discussed as rewetting of peatlands have been proposed as a tool to reduce climate impacts (Günther et al., 2020). However, some studies have shown that rewetting or restoring peatlands in forested areas in Scandinavia potentially increase the DOC load downstream (Nieminen et al., 2020, 2021). Knowledge about quality and biodegradability of the released carbon are crucial in understanding the fate of DOC in aquatic systems. Brownification is primarily driven by refractory organic substances, sometimes referred to as humic substances, which are generally resistant to biological degradation (Farjalla et al., 2009). However, it has been demonstrated that these substances can undergo biodegradation (Fasching et al., 2014) and that their molecules are susceptible to UV light (Granéli et al., 1996). Water bodies containing an abundance of humic substances might not be as productive, even though they possess high nutrient concentrations. This is largely due to the fact that these nutrients are predominantly incorporated into organic matter (Drakare et al., 2003), which can influence biological activity and the subsequent breakdown processes.

Given the widespread issue of browning in freshwater ecosystems, which results from increasing DOC and iron concentrations, it is crucial to find solutions. Constructed wetlands offer a potential solution on the browning problem. However, research on how wetlands impact browning remains limited (Borgström et al., 2024). Previous studies have highlighted a lack of comprehensive investigations into the watercolor‐reducing effects of wetlands, both natural and constructed (Djerf et al., 2023). Although field experiments are valuable, controlling all relevant factors can be challenging. Additionally, if wetlands are not specifically designed for research purposes, diffuse inflow and outflow—such as groundwater infiltration—may occur. Mesocosms provide an alternative method to explore wetland degradation potential. In this study, a similar experimental setup to that of Svensson et al. (2015) is adopted, with a notable difference: instead of using stormwater, naturally brown water is used as the inflow source.

The aim of this study was to investigate the potential reduction of naturally colored water in constructed wetlands, with a specific focus on the effects of residence time. This focus aimed to enable the estimation of the area of constructed wetlands necessary for achieving efficient reduction. Unlike previous studies, for example, (Djerf et al., 2023) the research was conducted in a more controlled environment, allowing better control over factors such as inflow and outflow and ensuring a consistent retention time.

## MATERIAL AND METHOD

This study employed six outdoor mesocosms, each with a volume of one cubic meter, to simulate surface flow wetlands. The mesocosms were set up following a similar approach as Svensson et al. (2015), but with a key difference: Naturally brown water from a nearby river was used as the inflow source. From the results by Svensson et al. (2015), it was expected that retention time would need to be long; thus, 7 and 14 days were chosen for this study.

### Experimental setup

The study site was situated on the bank of Bivarödsån, a tributary of the Helgeån River in Sweden (WGS84 coordinates: 56°10′6.6”N, 14°9′49.1″E). Bivarödsån has been considerably affected by brownification, resulting in a 315% increase in water coloration from a yearly mean of 69 mg/L PT in 1976 to 286 mg/L PT in 2021, as recorded by the environmental survey (SLU, 2024).

**FIGURE 1:**
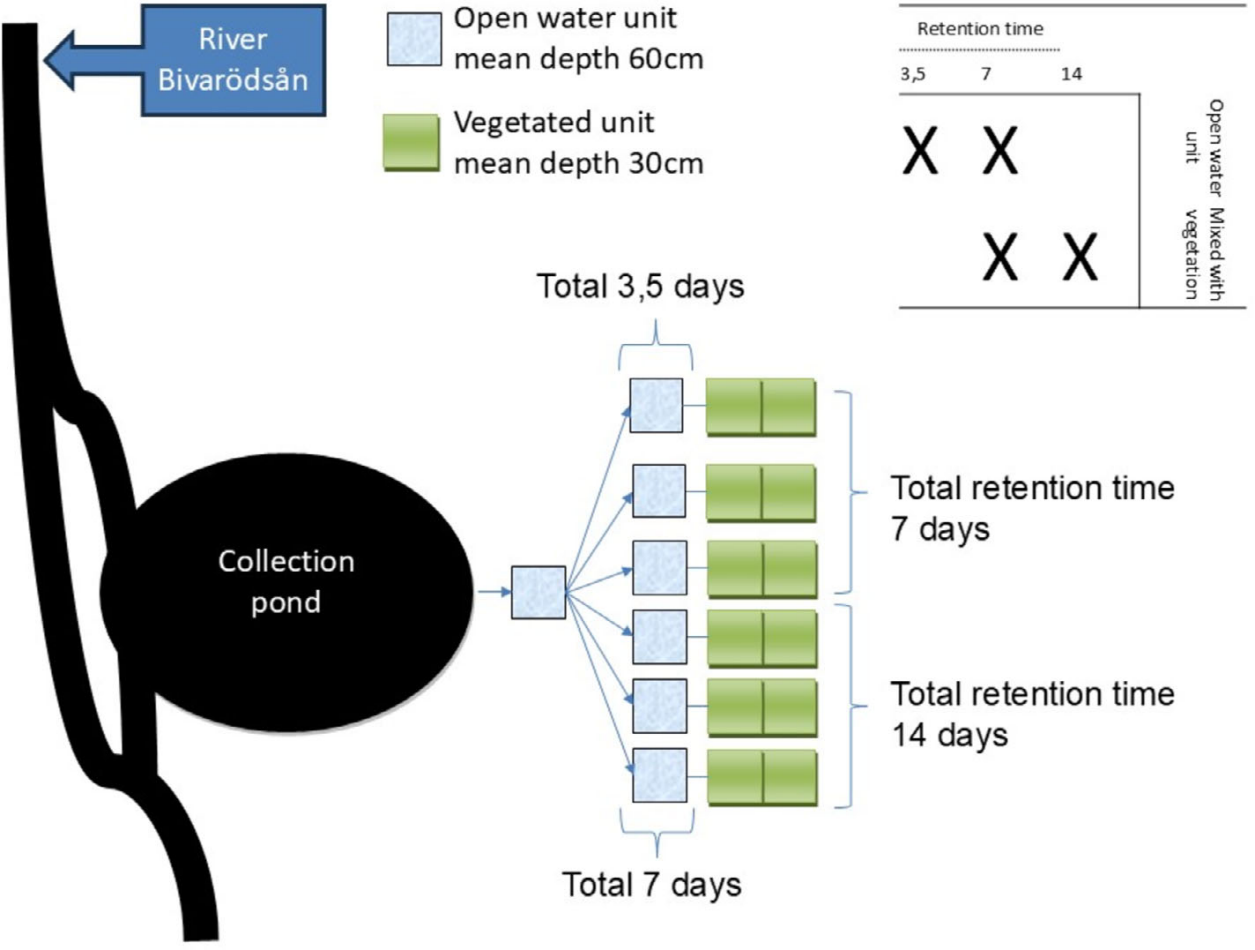
Experimental wetland design with four treatment combinations and three replicates used in this study.

The experiment spanned 3 years (2020–2022), with the initial year focused on establishing a vegetated ecosystem. Each mesocosm comprised three tanks: one for open water and two shallower, vegetated tanks (see Figure 1). This arrangement allowed for testing four different treatments in triplicate. Specifically, three mesocosms had a 7‐day retention time, while three others had a 14‐day retention time. Sampling was conducted at three locations: inflow, post‐open water, and outflow, resulting in four distinct treatment conditions (see Figure 1).

Each part of the mesocosm unit had a volume of 600 L. The surface area of the open water unit was 1 m^2^, resulting in a mean water depth of 60 cm. The vegetated unit had a surface area of 2 m^2^, with a mean water depth of 30 cm. This vegetated unit consisted of two tanks connected in series. To ensure proper water mixing, the inlet was positioned above the substrate surface on the bottom, and the outlet was designed as an overflow. Three mesocosm systems were fed with an inflow of 170 L/day, while the other three received 85 L/day, resulting in hydraulic retention times (HRTs) of 7 and 14 days, respectively. Water was pumped from the river to a separate tank using a submersible water pump on a timer, delivering fresh water every third hour. From this tank, the mesocosms were supplied with water using more accurate peristaltic pumps (520SN, Watson‐Marlow, Falmouth, UK). The pumping operation continued each year until winter, when water temperatures approached 0°C, and the pipes would freeze and burst. During the winter period, the tanks were filled with water but had no flow through them. Each year, the operation started in early April and ended by early November, leaving the system inactive for 5 months due to local climate conditions.

**FIGURE 2:**
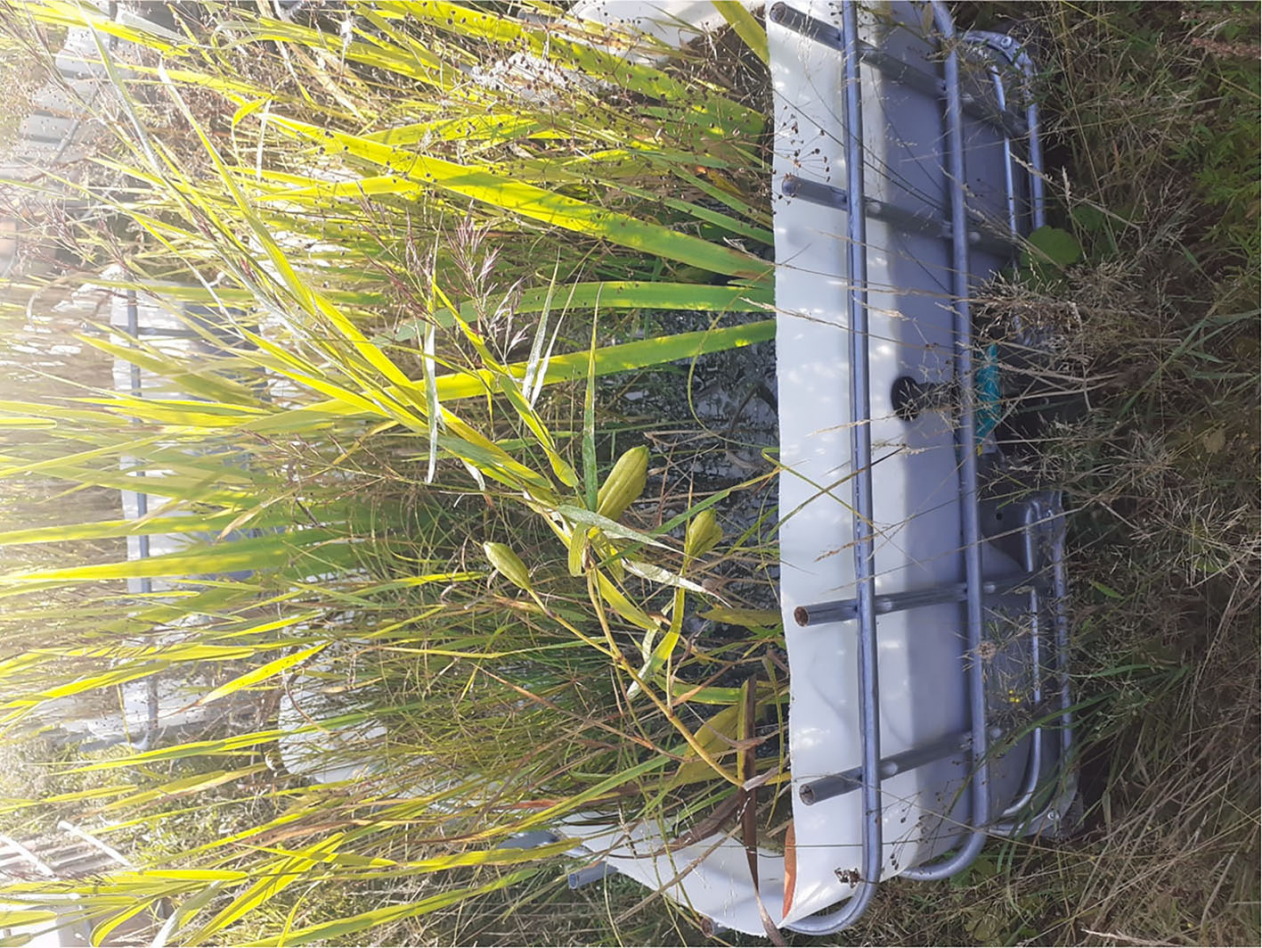
Showing one of 12 vegetated mesocosms units after 2 years.

The vegetated units were built using a 10 cm layer of crushed granite and a 10 cm layer of Lake Ivösjön sediment. To ensure rapid plant establishment, the mesocosm was planted with rhizomes of common reed (*Phragmites australis*) and seedlings of yellow iris (*Iris pseudacorus*) and bulrush (*Typha latifolia*). The organic‐rich sediments collected from the lake shore, when plants and rhizomes were harvested, served as the planting medium. Over 2 years, various plants colonized the units, with *Phragmites* and *Iris* becoming dominant (see figure 2).

### In situ measurements and sampling

In situ measurements of pH, electrical conductivity, dissolved oxygen, and temperature were taken using the Hach Lange HQd portable meter. All in situ measurements were made 15 cm from the mesocosm surface. Water samples were collected and promptly analyzed in the University's laboratory on the same day, except for iron samples, which were stored frozen. All samples were filtered through a 0.45 μm filter (MCE membrane, Millipore). Total DOC concentrations were analyzed using the Hach Lange test kit LCK 386 (according to the standard method EN 1484, DIN 38409‐H3) after filtration. Water color was measured in a 5 cm cuvette at λ420 nm following the Swedish standard (SS‐EN ISO 7887). True color was measured after filtering with a 0.45 nm filter, while apparent color was measured without filtration at the same wavelength.

During the 2‐year experimental period, samples were collected every 14th day. Before each sampling period, the pumps ran for 28 days in the units with low retention time and 14 days in the higher retention time units to ensure proper water circulation before sampling. The same practice was followed if there was an interruption in the flow in one or more of the mesocosms. Interruptions sometimes occurred during the experiment due to causes like summer drought or rodents.

### Data analyze

Treatment performances were calculated in percent as the difference in inflow concentration divided by the inflow value. For example, $\frac{\text{Inflow}-\text{outflow}}{\text{Inflow}}=\text{Reduction}\%.$

Statistical analyses were performed in GraphPad Prism (version 10.00.3) using linear regression and two‐way ANOVA. Gaussian normality tests with Shapiro–Wilk test were conducted before data usage. Further analysis employed two‐way ANOVA (compeering each group with every other group) combined with Tukey's post hoc tests. A *p*‐value of 0.05 was considered significant.

## RESULTS AND DISCUSSION

### Water characteristics

The water in the river Bivarödsån that was used in the mesocosm was highly colored, with a mean filter color absorbance of 0.62, corresponding to 310 mg/L PT (see Table 1) as yearly mean. The DOC was recorded at 31.4 mg/L throughout the year, and iron levels were similarly elevated, measured at 2.6 mg/L. Furthermore, the water was neutral, with a mean pH of 6.7, and well‐oxygenated, with a mean oxygen concentration of 9.0 mg/L. Notably, the variation (CV) in the measured parameters was low (less than 30%) throughout the year, showing that there is no significant seasonal variation in this catchment for the measured parameters.

**TABLE 1:** Characteristics of inflowing water to the mesocosm for the years 2021 and 2022, described with average values, standard deviation, coefficient of variation, and the number of samples.

	pH	Oxygen (mg/L)	Oxygen (%)	EC μS/cm	Temp °C	Color (apparent color) Abs 420	Color (true color) Abs 420	DOC mg/L	Iron mg/L
Average	6,7	9,0	81,7	109,1	11,3	0,8	0,62	31,4	2,6
Sd	0,4	1,8	16,4	16,9	4,2	0,2	0,16	5,6	0,7
CV	6%	20%	20%	16%	37%	23%	26%	18%	28%
n	26	22	22	24	20	26	26	26	26

Abbreviation: DOC, dissolved organic carbon.

Inflowing water was characterized on nutrient content twice a year at an accredited commercial laboratory and are presented in Table 2. According to local environmental regulations, the mesocosm's inflowing water is high in phosphorus but low in total nitrogen. As expected, most of the nutrients are not in soluble form and probably incorporated in organic matter. However, some phosphate and nitrate are in available form see Table 2

**TABLE 2:** Characteristics of inflowing water nutrient status to the mesocosm, made two times a year during the study period.

Parameter	Unit	14 Jun 2021	14 Sep 2021	9 may 2022	14 Oct 2022
Total nitrogen. N	μg/L	1400	1300	1200	1200
Nitrite nitrogen. NO2‐N	μg/L	<0.1	<1.0	<1.0	<0.1
Nitrate nitrogen. NO3‐N	μg/L	280	90	430	430
Total phosphorus. P	μg/L	51	61	47	42
Phosphate phosphorus. PO4‐P	μg/L	10	13	7.2	5.5
Iron. Fe	mg/L	3.6	4	3	2.5
Manganese. Mn	mg/L	0.22	0.2	0.23	0.15

**FIGURE 3:**
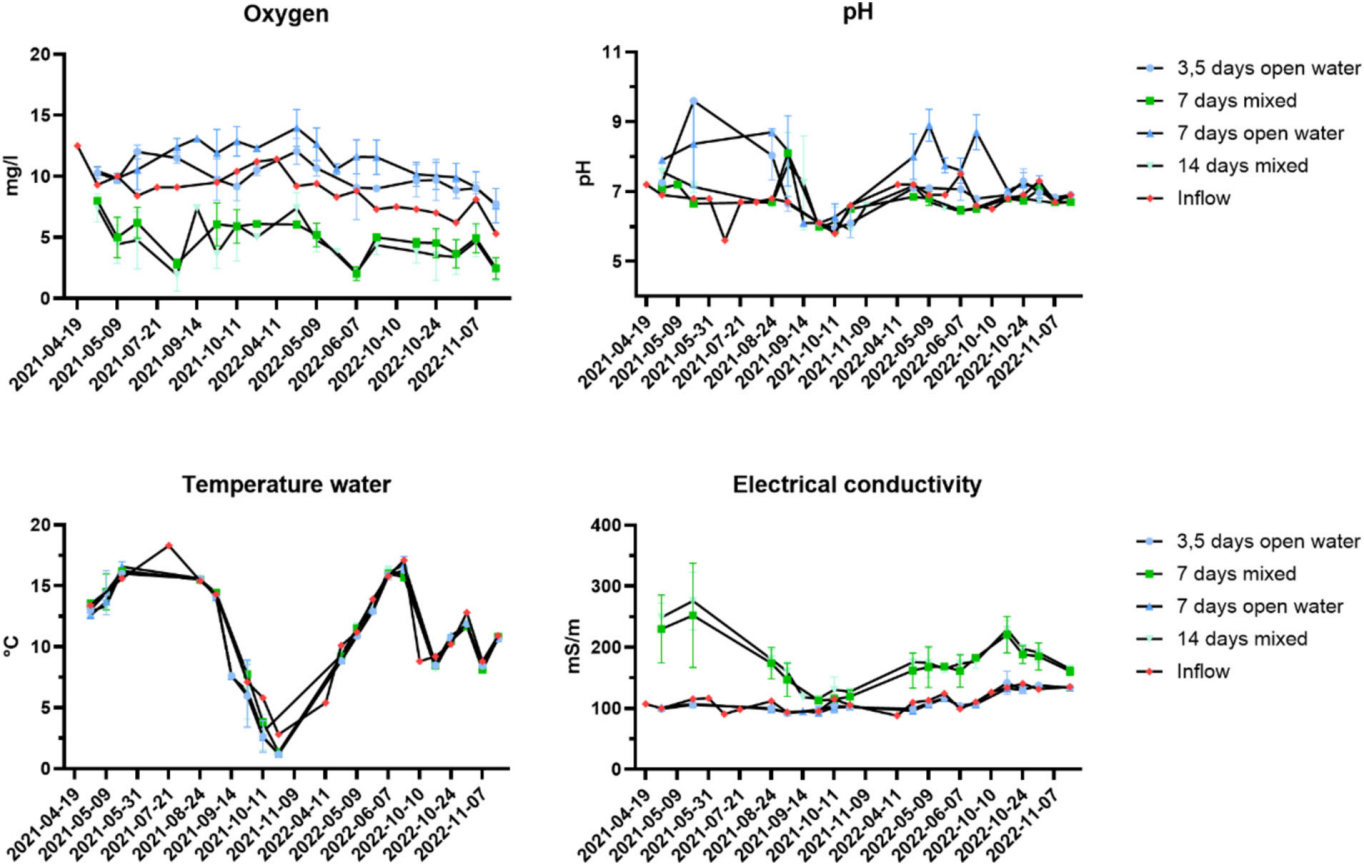
Temporal variation of water quality parameters: oxygen. pH. Temperature. And electrical conductivity during the monitoring period. Values presented are mean values (*n* = 3) for each treatment with SD within brackets.

The water temperature varied from 1.2 to 18°C during the two seasons within the mesocosms, note that the sampling and pumping were shut down during winter, to avoid damaging the pumps. pH levels increased in the mesocosm with only open water compared to the vegetated systems during the warmer periods (see Figure 3). This variation could be attributed to the high photosynthetic rate, which removes dissolved carbon dioxide from the water. Also supporting this are the difference in oxygen between the inflowing water: The open water within sun‐exposed areas had a higher concentration of oxygen. While the mesocosm with vegetation had a lower concentration.

Additionally, variations in electrical conductivity were observed between the systems. Specifically, the mixed systems that incorporated both open water and vegetation exhibited higher electrical conductivity compared to systems with only open water (see figure 3). This increase in conductivity suggests that the lake sediment used as a plant substrate contributed ions, potentially supplying essential macro‐ and micronutrients to the vegetated systems.

### Color reduction

**FIGURE 4:**
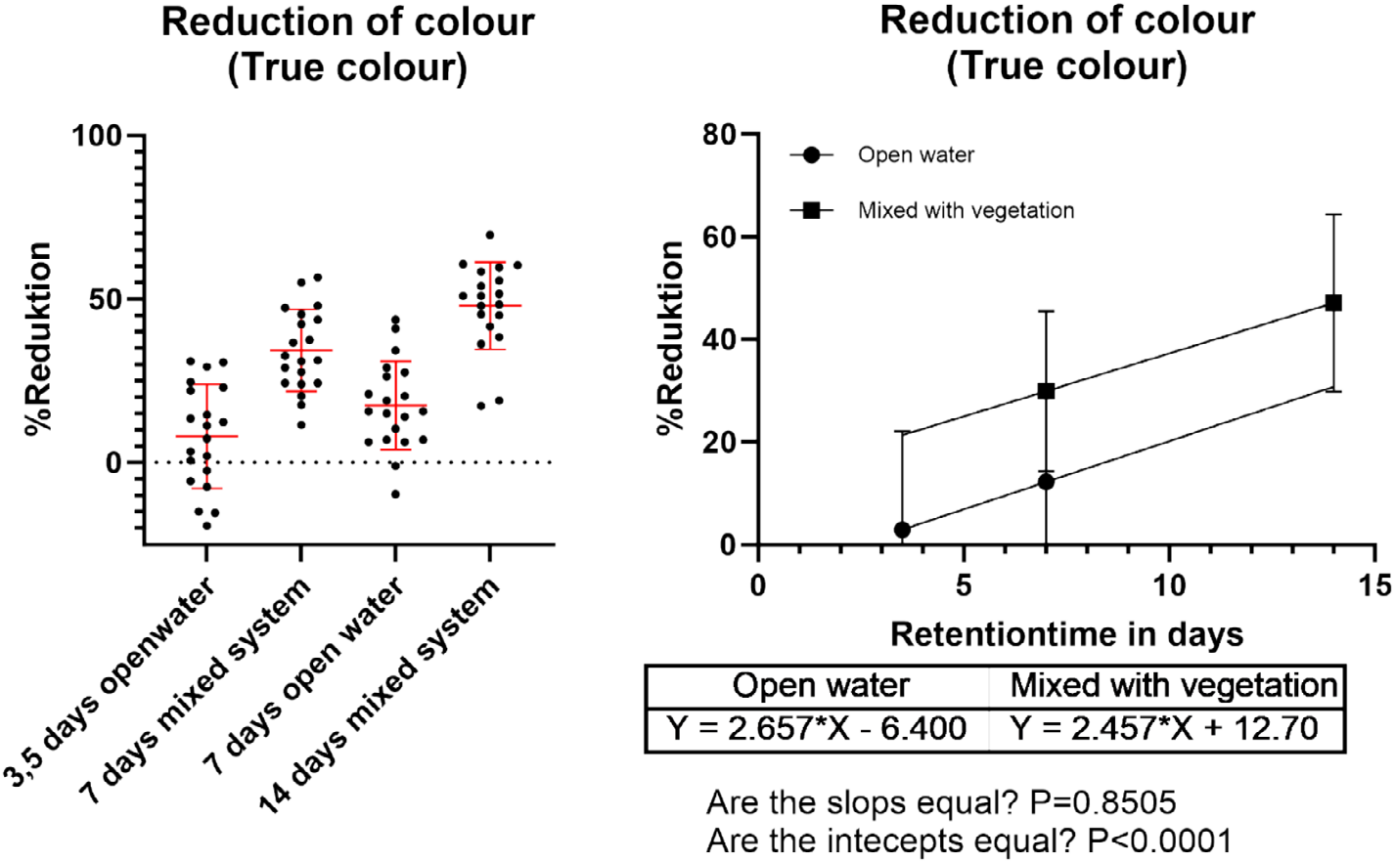
Describes the reduction of color within the mesocosm compared to the inflow. Left figure shows the reduction for the different treatments, and the right figure shows the reduction as a linear regression depending on the retention time.

After 14 days, color decreased by 47% (SD 17%), with an average daily reduction in the system of about 2.5%. The degree of color reduction in the mesocosm showed significant difference (*p* < 0.0001) between different treatments, such as open water versus mixed with vegetation. The mixed system proved to be the most effective in reducing color (see Figure 4). Nevertheless, linear regression analysis indicates that besides vegetation, residence time is also a crucial factor, showing increased reduction for both treatments based on residence time.

**FIGURE 5:**
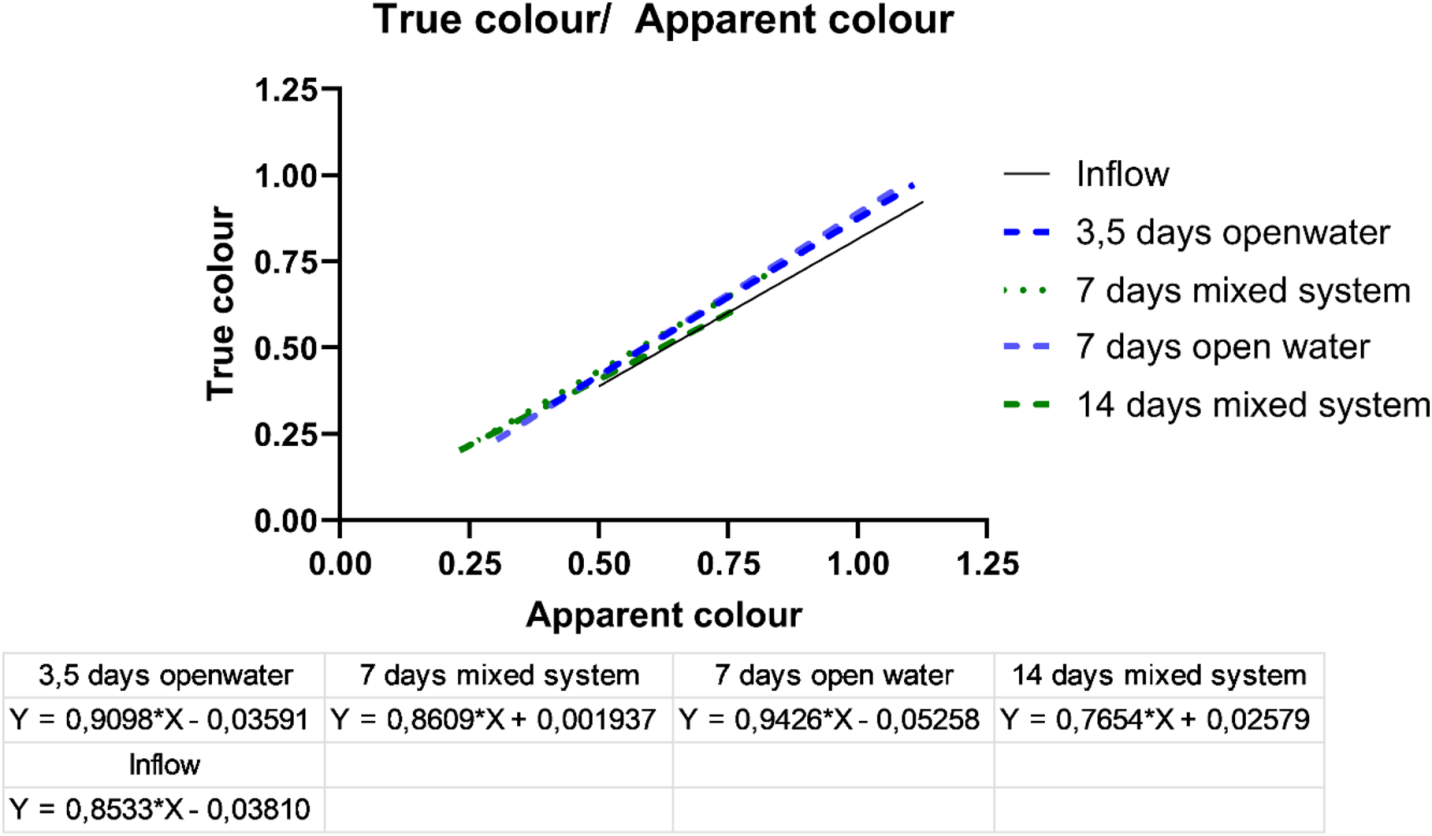
Plotting the true water color against the apparent water color.

The ratio of apparent water color to true water color helps determine if the wetland's color comes from dissolved substances or particulate matter. Interestingly, the color is mainly due to dissolved substances since there is only a minor difference between true and apparent color measurements (see Figure 5). When looking at retention times ranging from 3.5 to 7 days, we notice slightly more true water color (meaning fewer particles) compared to the inflow. However, in the 14‐day mixed system, there is a slight shift, with more apparent color observed in the mixed system than in the inflow. This means that particulate organic matter is higher and probably coming from decaying plants.

**FIGURE 6:**
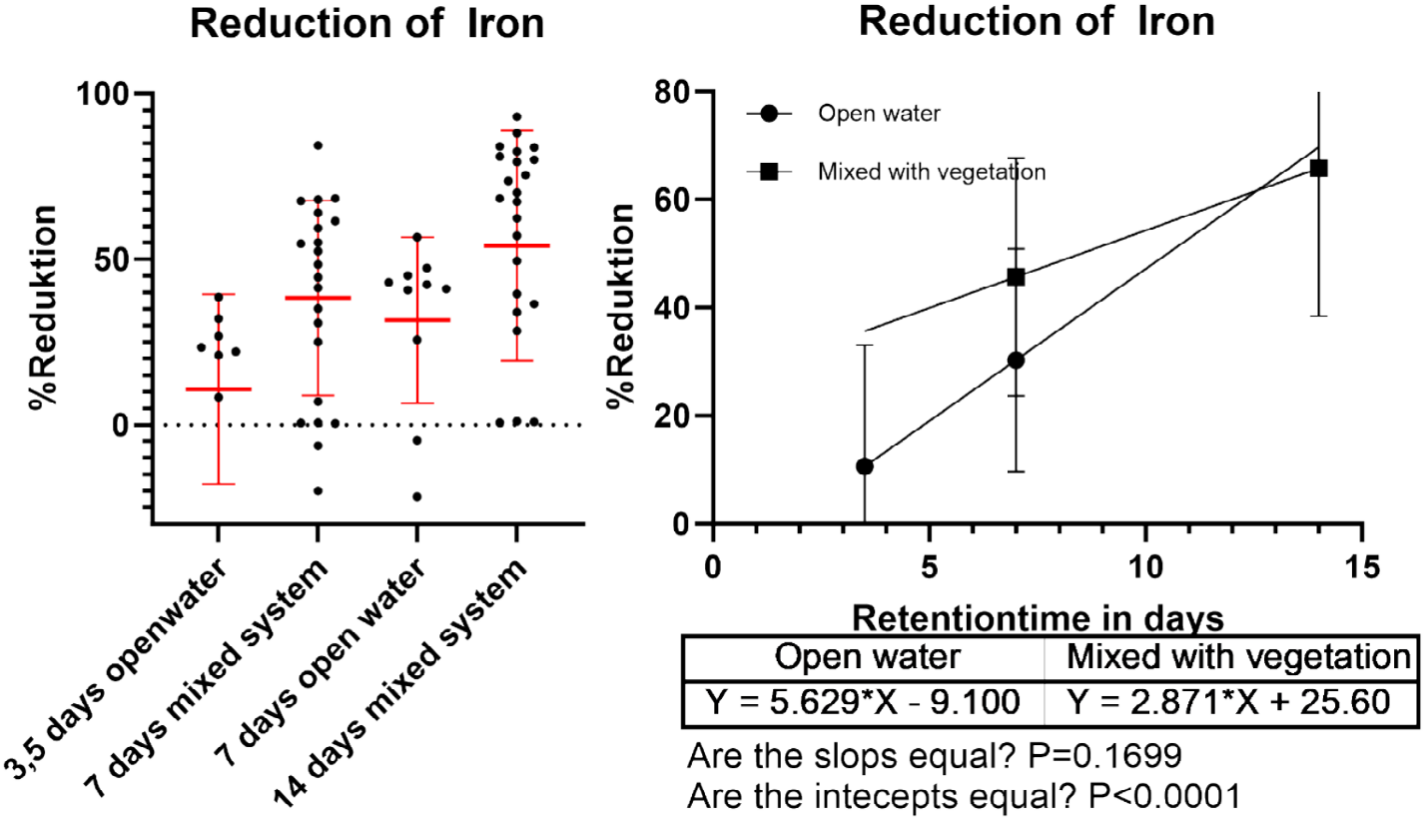
Describes the reduction of iron within the mesocosm compared to the inflow. Left figure shows the reduction for the different treatments, and the right figure shows the reduction as a linear regression depending on the retention time.

After 14 days, iron concentrations dropped by 66% (SD 27%), with a daily reduction averaging between 5.6% and 2.8% (Figure 6). The decrease was initially greater in the mesocosm with mixed systems but less pronounced over longer periods. Iron showed more significant variability than other parameters, possibly due to its solubility being influenced by oxygen levels and redox potential fluctuations, causing it to settle as solids and then dissolve again.

### DOC reduction

**FIGURE 7:**
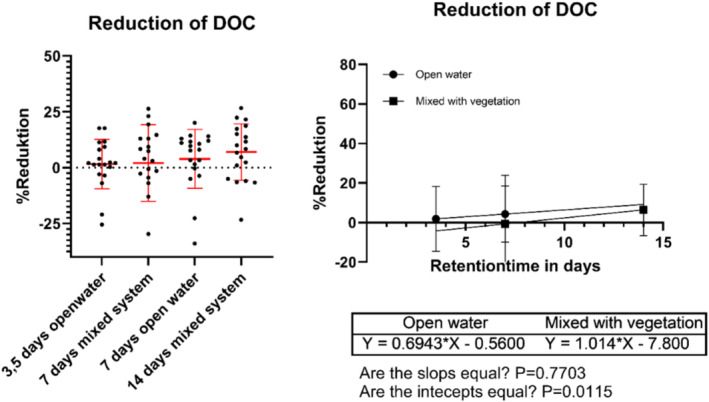
Describes the reduction of dissolved organic carbon (DOC) within the mesocosm compared to the inflow. Left figure shows the reduction for the different treatments, and the right figure shows the reduction as a linear regression depending on the retention time.

Noteworthy, there are significantly less reduction in DOC compared to reduction of color and iron in the study. The reduction in DOC is less pronounced compared to other measured parameters. After 14 days, there was a mean reduction of 6% (SD 13%) within the mesocosms. The analysis of variance (ANOVA) did not reveal any significant differences between treatments (see Figure 7).

The results indicate that in the mesocosms, both color and iron concentrations decrease, while DOC levels do not, suggesting no direct link between DOC concentration and color. This implies that color‐related molecules are reducing within the wetland, but different types of DOC are forming. It could also mean Fe/DOC complexes responsible for color are broken (Maloney et al., 2005; Xiao et al., 2013). This shows that contrary to previous research (Djerf et al., 2023) constructed wetlands could be utilized to reduce browning, also supported by other studies (Borgström et al., 2024).

While Borgström et al. (2024) emphasize photodegradation as a crucial process in humic substance breakdown, this study reveals an alternative mechanism in vegetated mesocosms. Despite the known challenges in decomposing humic substances and the anticipated role of UV light in their breakdown (Granéli et al., 1996), the results indicate that vegetated systems may employ alternative pathways for reducing color. The dense vegetation effectively shading the water appears to contribute to color reduction, independent of UV exposure. However, this result shows that color reduction and decrease in DOC are not interlinked, as the color reduction are more correlated to the iron decrease. Alternatively, the organic molecules causing the color may be reduced, but this is masked by the production of other organic molecules with different characteristics within the wetland.

It is widely acknowledged that wetland vegetation, particularly macrophytes, plays a crucial role in the removal of pollutants (Vymazal et al., 2021). However, the contributions of macrophytes are predominantly indirect. These include providing oxygen to the rhizosphere, offering substrate (roots and rhizomes) for the growth of attached bacteria, and serving as insulation during cold periods. This study was not designed to evaluate any specific macrophytes phytoremediation potential; however, the results show that the vegetation increases the reduction capacity. Possibly, the formation of a biofilm in the vegetated system—known to reduce humic substances—plays a role (Franke et al., 2013). Additionally, the presence of vegetation might enhance sedimentation and flocculation processes, as suggested by Weyhenmeyer et al. (2014), further contributing to the reduction.

### Impact of wetland age on efficiency

Wetlands are not only capable of reducing organic matter but are also capable of producing DOC, sometimes referred to internal loading or background production (Garcìa et al., 2010). Mattsson et al. (2009) show that wetland in northern colder climate generally produces more DOC than wetland found in warmer climate. As a constructed wetlands (CW) matures after its construction plants will colonize and grow, eventually dead plant parts will accumulate. Depending on the climate and water quality a border of peat will be established along the shores of the wetland. The bacterial breakdown of plant debris transforms particulate organic matter into a dissolved organic matter (Garcìa et al., 2010). These mesocosms are only 2–3 years old and may still experience more buildup than breakdown of dead plant materials. Therefore, the mesocosm's efficiency in reducing DOC may be overrated.

### Sizing of CW

The results show that a CW with a retention time of 14 days can reduce color by approximately 50%. However, it is important to stress that 14 days is considered a long retention time. According to the Swedish Meteorological and Hydrological Institute (Swedish Metrological and Hydrological Institute, 2024), the average annual runoff in the Bivarödsån catchment area is 250 mm/m^2^ or 2500 m^3^/ha. Simplifying this to assume constant runoff throughout the year, it equates to a daily runoff of 6.9 m^3^/ha/day. To design a CW with a 14‐day retention time, the wetland must be capable of storing 96 m^3^ (6.9 m^3^/ha/day × 14 days). Therefore, with an average water depth of 0.5 m, the required wetland area would be 190 m^2^/ha. Since 190 m^2^ is 1.9% of 1 ha, 1.9% of the catchment area would need to be designated as wetlands to achieve a 50% reduction in color by this method.

### Comparison with previous studies

This study shows that the color is decreased to a greater extent than DOC within the mesocosms. Noteworthy, as the color is decreased, so is the iron concentration. The amount of iron concentration seems to have a higher correlation to the color reduction than DOC. However, as previously pointed out the inflowing organic material might not be the same as the outflowing organic material. Nevertheless, the connection to iron and color is in line with previously studies (Kritzberg & Ekström, 2012; Weyhenmeyer et al., 2014).

Previous studies suggested that wetlands need a long retention time to reduce the color in wetlands (Djerf et al., 2023; Svensson et al., 2015); thus, this study used a long retention time. Fourteen days is a long time, and wetlands with this retention time would have to be placed far upstream in the watershed, as the flow per area wetland then will be lower. Therefore, CW might be part of a solution but not the only solution for the brownification problem, and a different land use is needed to reduce the DOC to be released to the water from the beginning.

In this study, the mesocosm experiment was conducted under controlled conditions, with a setup aiming to mimic natural conditions as closely as possible. Thus, the mesocosms were planted 1 year in advance to create a credible wetland condition. The mesocosms were placed adjacent to a river affected by brownification to feed the mesocosms with inflow water. Mesocosms thus provide good control of retention time, and no unaccounted inflow from, for example, groundwater is skewing the results. However, mesocosms also have limits; since they were placed elevated from the ground, they have more area affected by sun and wind, and as they are small and have less mass, they exhibit a higher temperature variation than a wetland. This might limit the applicability of the results to real‐world scenarios, as temperature affects microbial activity and the decomposition rate of organic matter.

## CONCLUSION

This study assessed the effectiveness of CW in mitigating water browning by reducing water color, iron, and DOC concentrations in highly colored natural water. The findings demonstrate that vegetated CW reduced water color, with iron removal identified as a key driver. In contrast, DOC reductions were modest at.

Several additional key findings emerged from this study. The reduction in water color was strongly correlated with iron removal. Vegetated systems performed better than open water systems, likely due to enhanced sedimentation and biological activity in vegetated wetlands. These mechanisms suggest that the design of CW should prioritize vegetation and optimize retention time for maximal efficiency.

This research contributes to the growing body of knowledge on wetland ability as a tool for addressing freshwater browning. However, given the long retention times required for substantial color reduction, wetlands alone may not suffice to mitigate water browning at larger scales. Integrated approaches, including changes in land‐use practices and upstream management to reduce Iron and DOC inputs, will be essential for comprehensive solutions. Future studies should investigate the molecular composition of organic matter entering and exiting wetlands, focusing on biodegradability and the role of iron‐DOC complexes. Additionally, scaling up these findings to landscape‐level applications and assessing their cost‐effectiveness will be crucial for practical implementation.

## CONFLICT OF INTEREST STATEMENT

The authors declare no conflict of interest.

## DECLARATION OF GENERATIVE AI AND AI‐ASSISTED TECHNOLOGIES IN THE WRITING PROCESS

During the preparation of this work, the author used Microsoft Bing Copilot to improve language and spelling. After using this tool/service, the author reviewed and edited the content as needed and take full responsibility for the content of the published article.

## Data Availability

The data that support the findings of this study are openly available in Mesocosm: ett verktyg för att validera konstruerade våtmarke at https://doi.org/10.5878/4jmy-v604.
